# Age- and sex-disaggregated disease burden among the older persons in India

**DOI:** 10.1186/s12877-024-05614-w

**Published:** 2024-12-19

**Authors:** G Anil Kumar, Anamika Pandey, Sailesh Mohan, Dorairaj Prabhakaran, Rakhi Dandona

**Affiliations:** 1 Public Health Foundation of India, House No. 60, 4th Floor, Lane 2, Part of Saidulajab Extension, Near Saket Metro Station Gate No. 2, New Delhi, India; 2 Centre for Chronic Disease Control, C1/52, 2nd Floor, C1/52 New Delhi, Safdarjung Development Area 110016, India; 3 London School of Hygiene & Tropical Medicine, Keppel Street, London, WC1E 7HT UK; 4 Institute for Health Metrics and Evaluation, 2301 Fifth Avenue, Suite 600, Seattle, WA 98121 USA

**Keywords:** Disease burden, Elderly, Gender, Global burden of disease study, Healthy ageing, India, Injury, Non-communicable diseases, Older persons, Policy, Program, Service provision

## Abstract

**Background:**

In the context of the increasing number and proportion of population aged 60 years or more in India, it is imperative to understand their health needs for ensuring healthy ageing.

**Methods:**

Using data from the Global Burden of Diseases, Injuries, and Risk Factors Study (GBD) 2019, we identified the top ten causes of disability-adjusted life years (DALYs), years of life lost (YLLs), and years lived with disability (YLDs) disaggregated by sex and age groups (60–64 years, 65–69 years, 70–74 years, 75–79 years, and ≥ 80 years) for India in 2019. We analysed the proportional contribution of individual causes to the total DALYs due to communicable diseases (CMNNDs), non-communicable diseases (NCDs), and injuries disaggregated by age and sex. We report the state-level heterogeneity in the crude DALY rate for CMNNDs, NCDs, and injuries for older persons disaggregated by sex. Additionally, we reviewed if the data capture of service delivery indicators on older persons were age- and sex-disaggregated in the Health and Wellness Centres (HWCs), and in the National Programs aimed at the Health Care for the Elderly (NPHCE), Prevention and Control of Non-communicable Diseases (NP-NCD), Control of Blindness and Visual Impairment (NPCBVI), Prevention & Control of Deafness (NPPCD), the Mental Health Program (NMPH), and the AYUSH Musculoskeletal Disorders Program (MSDP) within the context of disease burden.

**Results:**

The older persons accounted for a total of 136.1 million DALYs (29.1% of the total DALYs) in 2019 of which 77.9% were from NCDs, 14.8% from CMNNDs, and 7.3% from injuries, and nearly two-thirds of DALYs were accounted by YLLs. In NCDs, cardiovascular diseases, chronic respiratory diseases, neoplasms, diabetes and kidney diseases, and musculoskeletal disorders accounted for nearly 80% of DALYs for both sexes. There were variations in the magnitude of disease burden by specific diseases and conditions between females and males, and by age groups within both sexes particularly for injuries and CMNNDs. Injuries accounted for more YLDs than YLLs, ranging between 5.9%-15.2% for females and 15.3%-17.3% for males, with the females having a higher contribution to total injury related DALYs due to falls as compared to the males (54.4% vs 36.6%), whereas the males had a higher contribution to total DALYs due to road injuries (33.8% vs 19.4%). There was substantial variation in the crude DALY rates of major disease groups by the two sexes across the states of India in 2019. The crude DALY rate for CMNNDs varied between 3.6 times -3.7 times between the states for females and males, respectively; NCDs varied between 1.3 times -1.9 times, and injuries varied 2.0 times -1.7 times. The capture of service utilisation indicators was not age- or sex-disaggregated in NPHCE, NPCBVI, NMHP, MSDP, and HWCs; sex-disaggregation was available in NP-NCD but not age-disaggregation; sex-disaggregated data was available for many service indicators in NPPCD but with no age disaggregation beyond 50 years and more. Only NP-NCD and NPPCD allowed for data capture by disease/condition or severity of disease/condition for the older persons whereas the other programs including NPHCE did not allow for much disaggregated understanding by the type of services availed.

**Conclusions:**

This comprehensive assessment of the differentials in disease burden among older persons across age, sex and states of India, and the gaps identified in the service utilisation data capture by age and sex for the older persons in the national health programs can provide crucial inputs for strengthening the on-going public health policy and programmatic efforts aimed at improving the health and well-being of the growing older population in India.

**Supplementary Information:**

The online version contains supplementary material available at 10.1186/s12877-024-05614-w.

## Introduction

All countries are expected to see a substantial increase in the number and the proportion of older population by 2030, with faster growth projected in low- and middle-income countries than in the high-income countries [1]. To foster healthy ageing and improve the lives of older people, the World Health Organization (WHO) is leading the United Nations Decade of Health Ageing 2021–2030, a global collaboration that brings together governments, civil society, international organizations, professionals, academic or research institutions, the media and the private sector to improve the lives of older people and their families and communities [2].

The proportion of people aged 60 years or more in India has increased from 6.1% in 1990 to 10.1% in 2020, and is projected to increase to 347 million by 2050, outnumbering the 0–14 years population by 2046 [3]. India launched the National Program for Health Care of the Elderly (NPHCE) in 2010 to provide better access to promotional, preventive, curative and rehabilitative services to the older persons, improve referral system when needed, and to increase the capacity of health care providers and community to provide health care to the older persons [4]. The NPHCE is administered under the aegis of the non-communicable diseases (NCD) cell within the health system, which has the mandate to implement the activities under the National Program for Prevention and Control of NCDs (NP-NCD) [5]. Knowledge of disease burden among the older persons and monitoring of health service utilisation by them is needed to provide for appropriate health services as well as to drive the national and local actions for healthy ageing. Furthermore, because the older persons are not a homogeneous group, data on the disease burden disaggregated by age and sex is imperative to better understand the specific issues affecting health and well-being of the older persons as they age. In this context, we present the disease burden among the older persons in India as estimated by the Global Burden of Diseases, Injuries, and Risk factors Study (GBD) 2019, [6] and assess the availability of age- and sex-disaggregation in the service utilization indicators for services provided to the older persons under a variety of relevant national health programs.

## Methods

We reviewed the definition of older persons used in India and globally [1, 7]. Older person is defined in India as individuals aged 60 years or above, which is lower than the age criteria of 65 years or above used globally [7]. We present the findings disaggregated by five age groups from 60 years onwards as available in the GBD Study – 60–64 years, 65–69 years, 70–74 years, 75–79 years, and 80 years or more – to provide a more nuanced understanding of the health needs of older persons in India.

We accessed the GBD Study 2019 data from the Institute for Health Metrics and Evaluation’s Global Health Data Exchange on 5 December 2023 [6]. The accessed data were the absolute number and count per 100,000 people for years of life lost (YLLs), years lived with disability (YLDs), and disease burden measured as disability-adjusted life years (DALYs) for females and males in the 5 age groups as indicated above for India and its states in 2019, for causes relating to communicable, maternal, neonatal, and nutritional diseases (CMNNDs), NCDs, and injuries. A comprehensive description of the metrics, data sources, and statistical modeling for disease burden and risk factor estimation in the GBD Study 2019 has been provided elsewhere [8–11].

The major data sources used in the GBD for cause-specific mortality estimation in India were verbal autopsy from the Sample Registration System, medically certified causes of deaths, cancer registries, and smaller verbal autopsy studies [11]. The major input data sources used in the GBD to quantify the non-fatal burden of disease in India were representative population-level surveys and cohort studies, program-level data on disease burden from government agencies, surveillance system data on disease burden, administrative records of health-service encounters, disease registries, and a wide range of other studies done across India [11]. These studies included published literature as well as unpublished studies that were identified and accessed through a network of expert group members and collaborators in India. DALYs, a summary measure of total health loss, are available for India and states in the GBD by summing YLLs and YLDs for each cause, age, and sex [8].

The accessed data was downloaded in CSV format as available from Global Health Data Exchange [6]. The data from CSV files were copied in MS-Excel to generate the proportions presented in this analysis. We analysed the percentage contribution of disease categories to total DALYs due to CMNNDs, NCDs, and injuries by five age groups and sex for India in 2019 using the GBD Study data, and assessed the proportional contribution of YLLs and YLDs in total DALYs due to disease categories with CMNNDs, NCDs and injuries. We present the top 10 individual causes of DALYs, YLLs and YLDs disaggregated by sex and by five age groups to highlight the differential fatal and non-fatal burden on older persons health for India in 2019. The percentage contribution of each of the top 10 individual causes to all-cause DALYs, YLLs and YLDs is presented by age and sex. We also report on the contribution of major disease groups to total DALYs, YLLs and YLDs from the top 10 causes due to CMNNDs, NCDs and injuries disaggregated by sex and age group in India in 2019. In addition, we analysed the sub-national heterogeneity in the crude DALY rate of CMNNDs, NCDs, and injuries for older persons disaggregated by sex in India in 2019. These findings are reported for 31 geographical units in India: the 28 states, the union territory of Delhi, the union territories of Jammu & Kashmir and Ladakh (combined), and the other smaller union territories combined (Andaman and Nicobar Islands, Chandigarh, Dadra and Nagar Haveli, Daman and Diu, Lakshadweep, and Puducherry). We report all the estimates with 95% uncertainty intervals (UIs) where relevant. The UIs were based on 1000 runs of the models for each quantity of interest, with the mean regarded as the point estimate and the 2.5th and 97.5th percentiles considered as the 95% UI. QGIS was used for plotting DALY burden estimates across the states of India. The GBD Study complies with the Guidelines for Accurate and Transparent Health Estimates Reporting (GATHER) statement [8].

We assessed if the data capture of service delivery indicators on older persons were age- and sex-disaggregated in the NPHCE [7] and NP-NCD [5]. In addition, we also assessed this availability for the national programs that are likely to cater to the older persons, which included the National Program for Control of Blindness and Visual Impairment (NPCBVI), [12] National Program for the Prevention & Control of Deafness (NPPCD), [13] the National Mental Health Program (NMHP), [14] and the National Program for Prevention and Management of Osteoarthritis and other Musculoskeletal Disorders (MSDP) [15]. Lastly, we reviewed the availability of age- and sex- disaggregation for service delivery indicators captured under the Health and Wellness Centres (HWCs) which are set up by the Government of India to deliver comprehensive primary health care, which is universal and free to the users [16].

## Results

An estimated 136.1 million DALYs (29.1% of the total DALYs) were attributed to the older persons in India in 2019, with 72.7% of these accounted for by YLLs (see Supplementary Table 1, Additional File). By the disease category groups, 77.9% of the total DALYs were from NCDs, 14.8% from CMNNDs, and 7.3% from injuries. The point estimate of DALY rate per 100,000 population for CMNNDS was higher in females than in males, and for NCDs it was higher in males than females across all the age categories of older persons, whereas the point estimate of DALY rate for injuries was generally higher in males than in females, except for > 75 years where females had higher rate than males (see Supplementary Table 2, Additional File).

### Disease burden among older females

**Fig. 1:**
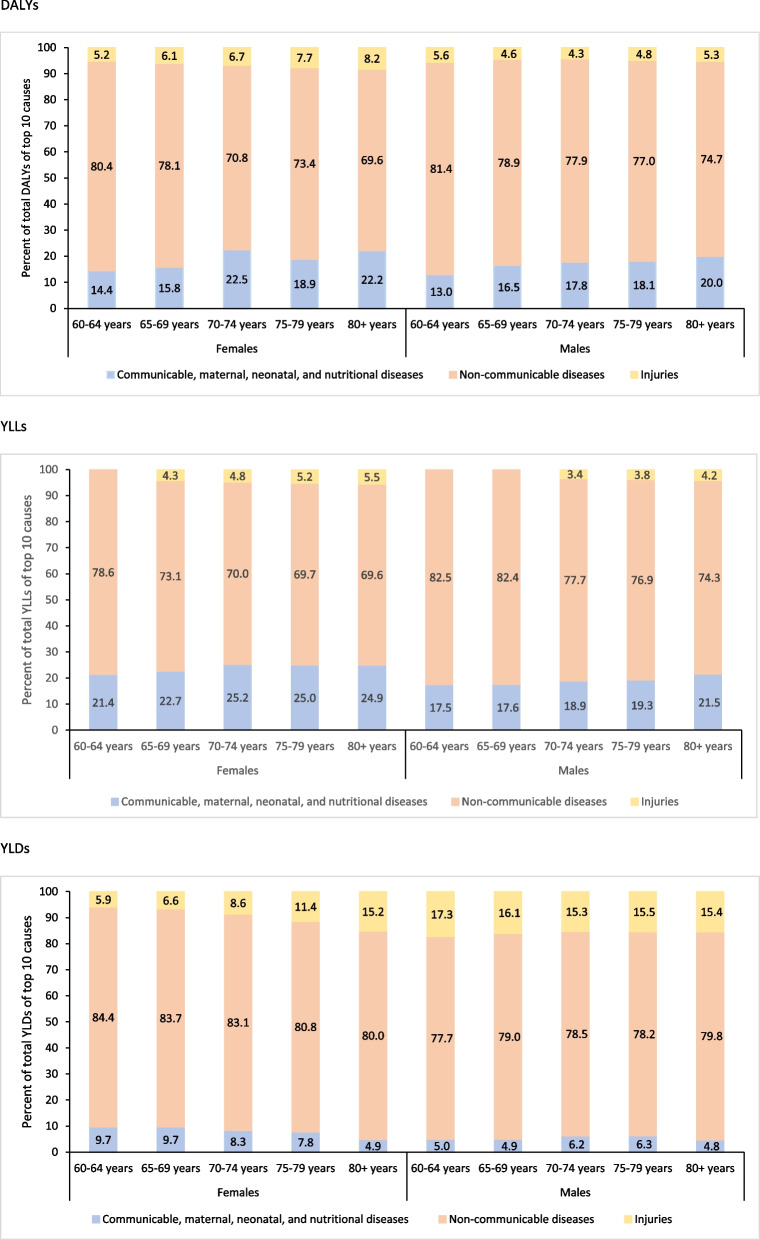
Percent contribution of major disease groups to the total disability-adjusted life years (DALYs), years of life lost (YLLs), and years lived with disability (YLDs) of top 10 causes in the population aged 60 years or more by sex in India in 2019, the Global Burden of Disease Study

Of the total estimated 67.6 million DALYs in older females in India in 2019, 76.3% were from NCDs, 16.3% from CMNNDs, and 7.3% from injuries; the YLLs accounted for 70% of the overall DALYs (see Supplementary Table 1, Additional File). The distribution of top 10 individual causes of DALYs, YLLs and YLDs for females by age groups are shown in Table 1, Fig. 1 and Supplementary Table 3 (Additional File). In all age groups except 70–74 years, seven out of the top 10 causes of DALYs were NCDs, contributing 80.4% of the total DALYs in 60–64 years, 78.1% in 65–69 years, 73.4% in 75–79 years, and 69.6% in 80 years or more. This pattern was similar when considering only the leading 10 causes of YLLs or YLDs, with 6–8 of the 10 leading causes being NCDs across all age groups (see Supplementary Table 3, Additional File). Injuries accounted for more YLDs than YLLs, and ranged from 5.9% in 60–64 years to 15.2% in 80 years or more (Fig. 1).

**Table 1:**
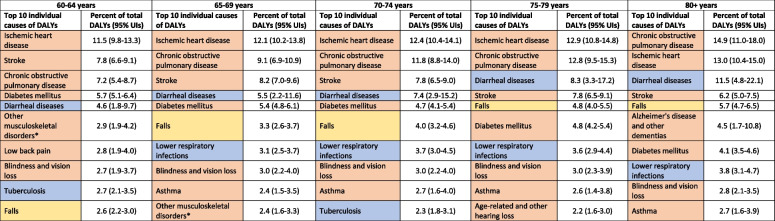
Top 10 causes of disability-adjusted life years (DALYs) among females 60 years and more in India in 2019, the Global Burden of Disease Study

*Other musculoskeletal disorders include lupus erythematosus, infectious arthropathies, inflammatory polyarthropathies, other joint disorders, systemic connective tissue disorders, deforming dorsopathies, spondylopathies, disorders of muscles, disorders of synovium and tendon, other soft tissue disorders, disorders of bone density and structure, osteomyelitis, other osteopathies, chondropathies, and other disorders of the MSK system and connective tissue

*UIs* Uncertainty intervals

Of the total CMNNDs DALYs in females, the highest contribution was from diarrheal diseases (44.7%) followed by lower respiratory infections (20.1%) and tuberculosis (14.0%) as shown in Supplementary Fig. 1 (Additional File). The contribution of diarrheal diseases increased with increasing age ranging from 32.3% in 60–64 years to 59.9% in 80 years or more, contribution of tuberculosis decreased with increasing age ranging from 19.1% in 60–64 year to 8.9% in 80 years or more, and the contribution of lower respiratory infections was similar across the age groups (see Supplementary Fig. 1, Additional File). Among females, YLLs accounted for majority of the DALYs due to tuberculosis (91.4%), lower respiratory infections (98.7%) and diarrheal diseases (94.0%), whereas YLDs accounted for all of the DALYs due to dietary iron deficiency (see Supplementary Fig. 4, Additional File).

Of the total NCD DALYs in females, the highest contribution was from cardiovascular diseases (31.4%) followed by chronic respiratory diseases (18.5%), neoplasm (10.3%), diabetes and kidney diseases (9.2%), and musculoskeletal disorders (7.4%) as shown in Supplementary Fig. 2 (Additional File). The burden of cardiovascular diseases and chronic respiratory diseases increased with increasing age ranging from 28.7% in 60–64 years to 33.0% in the highest age group and 12.9% to 25.0%, respectively; while that of neoplasms (range 13.2%—6.3%), diabetes and kidney diseases (10.3%—7.6%), and musculoskeletal disorders (10.1%—4.4%) decreased with increasing age (see Supplementary Fig. 2, Additional File). For females, YLLs accounted for majority of the DALYs due to neoplasms (98.0%), cardiovascular diseases (94.7%), chronic respiratory diseases (82.4%), digestive diseases (83.5%), neurological diseases (60.4%), and diabetes and kidney diseases (63.1%), and YLDs accounted for all of the DALYs due to sense organ disorders and 93.7% of the DALYs due to musculoskeletal disorders (see Supplementary Fig. 4, Additional File).

Of the total injury DALYs in females, the highest contribution was from falls (54.4%) followed by road injuries (19.4%) and other unintentional injuries (19.3%) as shown in Supplementary Fig. 3 (Additional File). The burden of falls increased with increasing age, with its contribution to total injury DALYs being 2.1 times higher in 80 years or more than 60–64 years age group (see Supplementary Fig. 3, Additional File). Whereas, the contribution of road injuries and other unintentional injuries decreased with increasing age ranging from 29.2% in 60–64 years to 9.8% in 80 years or more and 23.6% in 60–64 years to 13.3% in 80 years or more, respectively (see Supplementary Fig. 3, Additional File). Among females, YLDs accounted for a higher proportion of total DALYs for road injuries (65.4%), whereas YLLs contributed more to DALYs for self-harm (82.3%) and falls (58.6%) as shown in Supplementary Fig. 4 (Additional File).

### Disease burden among older males

Of the total estimated 68.5 million DALYs in males in India, 79.5% were from NCDs, 13.3% were from CMNNDs and 7.2% were from injuries; and YLLs accounted for 75.4% of the overall DALYs (see Supplementary Table 1, Additional File). The distribution of top 10 individual causes of DALYs, YLLs and YLDs for males by age groups are shown in Table 2, Fig. 1, and Supplementary Table 4 (Additional File). In all age groups except 60–64 years, six out of the top 10 causes of DALYs were NCDs, contributing 78.9% in 65–69 years, 77.9% in 70–74 years, 77.0% in 75–79 years, and 74.7% in 80 years or more. This pattern was similar when considering only the leading 10 causes of YLLs or YLDs, with 6–8 of the 10 leading causes being NCDs across all age groups. Injuries accounted for more YLDs than YLLs, with the highest contribution of 17.3% in 60–64 years, followed by 16.1% in 65–69 years and 15.5% in 75–79 years (Fig. 1).

**Table 2:**
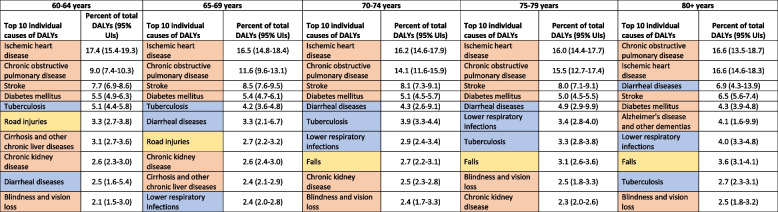
Top 10 causes of disability-adjusted life years (DALYs) among males 60 years or more in India in 2019, the Global Burden of Disease Study

*UIs *Uncertainty intervals

Of the total CMNNDs DALYs in males, the highest contribution was from diarrheal diseases (30.7%) and tuberculosis (30.0%), followed by lower respiratory infections (21.0%) (see Supplementary Fig. 1, Additional File). The contribution of diarrheal diseases and lower respiratory infections increased with increasing age ranging from 20.2% in 60–64 years to 44.5% in 80 years or more and from 16.0% in 60–64 years to 25.5% in 80 years or more, respectively. The contribution of tuberculosis decreased with increasing age, with its contribution to total CMNNDs DALYs being 2.4 times higher in 60–64 years (41.2%) than in 80 years or more (17.4%) as shown in Supplementary Fig. 1 (Additional File). Among males, YLLs accounted for majority of the DALYs due to tuberculosis (93.4%), lower respiratory infections (98.3%) and diarrheal diseases (91.2%), whereas YLDs accounted for all of the DALYs due to dietary iron deficiency (see Supplementary Fig. 4, Additional File).

Of the total NCD DALYs in males, the highest contribution was from cardiovascular diseases (34.7%) followed by chronic respiratory diseases (19.6%), neoplasms (10.8%), diabetes and kidney diseases (9.6%) as shown in Supplementary Fig. 2 (Additional File). The burden of chronic respiratory diseases increased with increasing age ranging from 14.6% in 60–64 years to 24.7% in 80 years or more; while the burden of neoplasms (range 12.6%−7.5%), diabetes and kidney diseases (range 10.3%−8.6%) decreased with increasing age and burden of cardiovascular diseases was similar across age groups (see Supplementary Fig. 2, Additional File). For males, YLLs accounted for majority of the DALYs due to neoplasms (98.3%), cardiovascular diseases (95.6%), chronic respiratory diseases (85.4%), digestive diseases (89.3%), neurological diseases (64.9%), and diabetes and kidney diseases (65.8%), and YLDs accounted for all of the DALYs due to sense organ disorders and 94.2% of the DALYs due to musculoskeletal disorders (see Supplementary Fig. 4, Additional File).

Of the total injury DALYs in males, the highest contribution was from falls (36.6%) and road injuries (33.8%) followed by other unintentional injuries (19.4%) as shown in Supplementary Fig. 3 (Additional File). The burden of falls increased with increasing age, with its contribution to total injury DALYs being 2.2 times higher in 80 years or more than in 60–64 years (see Supplementary Fig. 3, Additional File). Whereas, the contribution of road injuries and other unintentional injuries decreased with increasing age ranging from 40.7% in 60–64 years to 22.9% in 80 years or more and 20.7% in 60–64 years to 16.0% in 80 years or more, respectively (see Supplementary Fig. 3, Additional File). Among males, YLDs accounted for a higher proportion of total DALYs for road injuries (61.4%), whereas YLLs contributed more to DALYs for self-harm (95.2%) and falls (66.7%) as shown in Supplementary Fig. 4 (Additional File).

### Variation in disease burden across the states

**Fig. 2:**
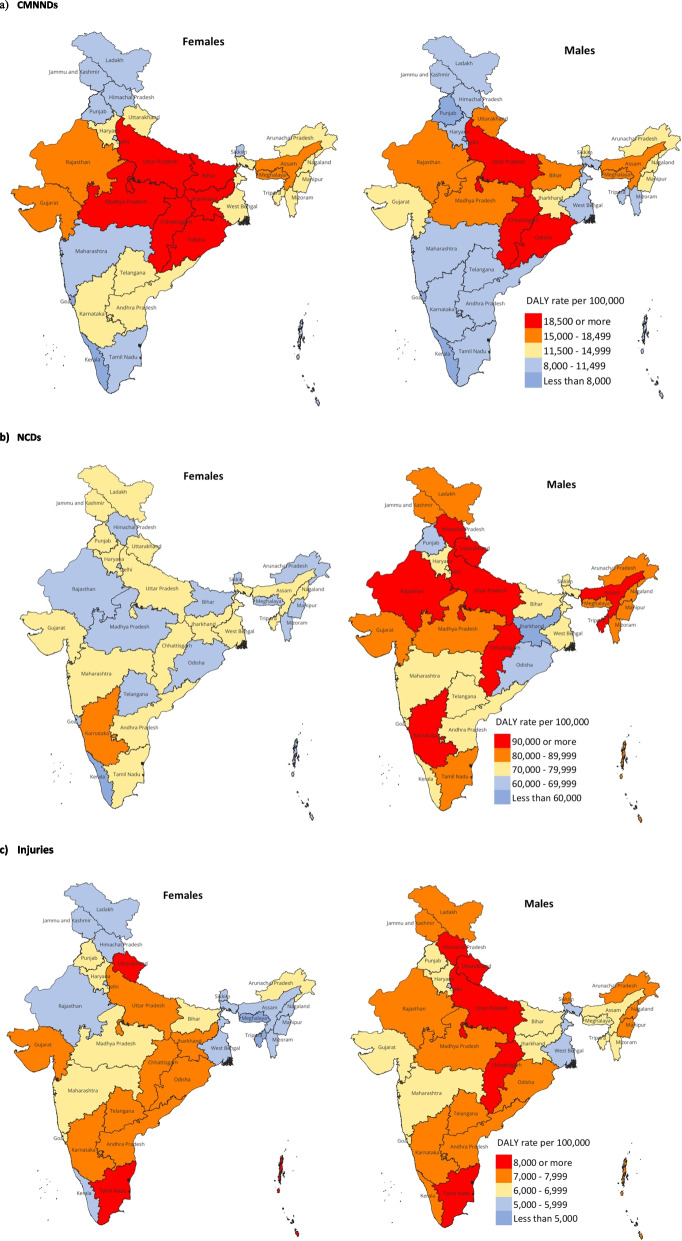
Crude DALY rate of communicable, maternal, neonatal, and nutritional diseases (CMNNDs), non-communicable diseases (NCDs) and injuries in females and males aged 60 years or more in the states of India in 2019, the Global Burden of Disease Study

There was substantial variation in the crude DALY rates of major disease groups by the two sexes across the states of India in 2019 (Fig. 2 and Supplementary Table 5, Additional File). For older females, the crude DALY rate for CMNNDs varied 3.6 times between the states ranging from 23,855 to 6,699 per 100,000 population, and for older males it varied 3.7 times ranging from 21,959 to 6,007 per 100,000 population (see Supplementary Table 5, Additional File). The Empowered Action Group (EAG) states of Uttar Pradesh, Chhattisgarh, Odisha, Bihar, Jharkhand, and Madhya Pradesh had the highest CMNNDs DALY rate in 2019 for females, whereas that for males were in Uttar Pradesh, Odisha and Chhattisgarh (Fig. 2a).

The crude DALY rate for NCDs varied 1.3 times between the states in 2019 for females ranging from 80,733 to 59,942 per 100,000 population, and for males it varied 1.9 times ranging from 1,04,972 to 55,818 per 100,000 population (see Supplementary Table 5, Additional File). The burden of NCDs was generally higher in males than females across the states (see Supplementary Table 5, Additional File). For males, Uttarakhand, Karnataka and Himachal Pradesh, and EAG states of Uttar Pradesh, Chhattisgarh, Rajasthan, and Assam had the highest DALY rate ranging from 91,131 to 104,972 per 100,000 population (Fig. 2b). For females, the state with the highest DALY rate of 80,733 per 100,000 population was Karnataka, with majority of the states having DALY rate ranging from 60,000 to 79,000 per 100,000 population (Fig. 2b and Supplementary Table 5, Additional File).

The crude DALY rate for injuries varied 2.0 times between the states ranging from 9,344 to 4,670 per 100,000 population for females in 2019, and varied 1.7 times for males ranging from 9,718 to 5,577 per 100,000 population (see Supplementary Table 5, Additional File). The highest burden of injuries for females was in the states of Tamil Nadu and Uttarakhand, and for males was in the states of Tamil Nadu, Uttarakhand and Himachal Pradesh and in the EAG states of Chhattisgarh and Uttar Pradesh (Fig. 2c).

### Age- and sex-disaggregation of service utilisation indicators

There was no age- or sex-disaggregation captured for the service delivery indicators in NPHCE (Table 3) whereas the sex-disaggregation was available for all but age-disaggregation for none of the indicators captured under NP-NCD (Table 4). The NPCBVI, NMHP, and MSDP do not capture service utilisation data disaggregated by either age or sex; whereas the NPPCD captured sex-disaggregated data for many service indicators but not all and age was captured only as 50 years or more (see Supplementary Table 6, Additional File). The HWCs (see Supplementary Table 7, Additional File) does not capture service utilisation disaggregated either by age or sex. Furthermore, only NP-NCD and NPCCD allowed for data capture by disease/condition or severity of disease/condition for the older persons (Table 4 and see Supplementary Table 6, Additional File) whereas the other programs including NPHCE did not allow for much disaggregated understanding by the type of services availed.

**Table 3:** Age- and sex- disaggregation in the available indicators for services provided to older persons under the National Program for Healthcare of the Elderly (NPHCE)

Services provided	Disaggregated by age	Disaggregated by sex
**Sub Centre**		
Number of elderly persons enrolled	No	No
Number of elderly persons checked up and provided health card	No	No
Number of elderly persons provided and used supportive devices	No	No
Number of home visits made	No	No
Number of elderly persons provided home-based care	No	No
Number of cases referred to Primary Health Centre/Community Health Centre	No	No
**Primary Health Centre**		
Geriatric clinic (once/week) held	No	No
Outpatient cases in Geriatric Clinics	No	No
Number of home visits made	No	No
Number of elderly persons provided home-based care	No	No
Number of elderly persons provided and used supportive devices	No	No
Cases referred to Community Health Centre/ District Hospitals	No	No
**Community Health Centre**		
Geriatric clinic (once/week) held	No	No
Outpatient cases in Geriatric Clinics	No	No
Number of home visits made	No	No
Number of elderly persons provided home-based care	No	No
Cases referred to District Hospitals/ Medical Colleges	No	No
**District Hospital**		
Outpatient cases in Geriatric Clinics	No	No
Inpatients admitted in Geriatric Ward	No	No
Cases referred to Medical Colleges/Regional Geriatric Centres	No	No
Geriatric camps organized at Community Health Centres/ Primary Health Centres	No	No

**Table 4:**
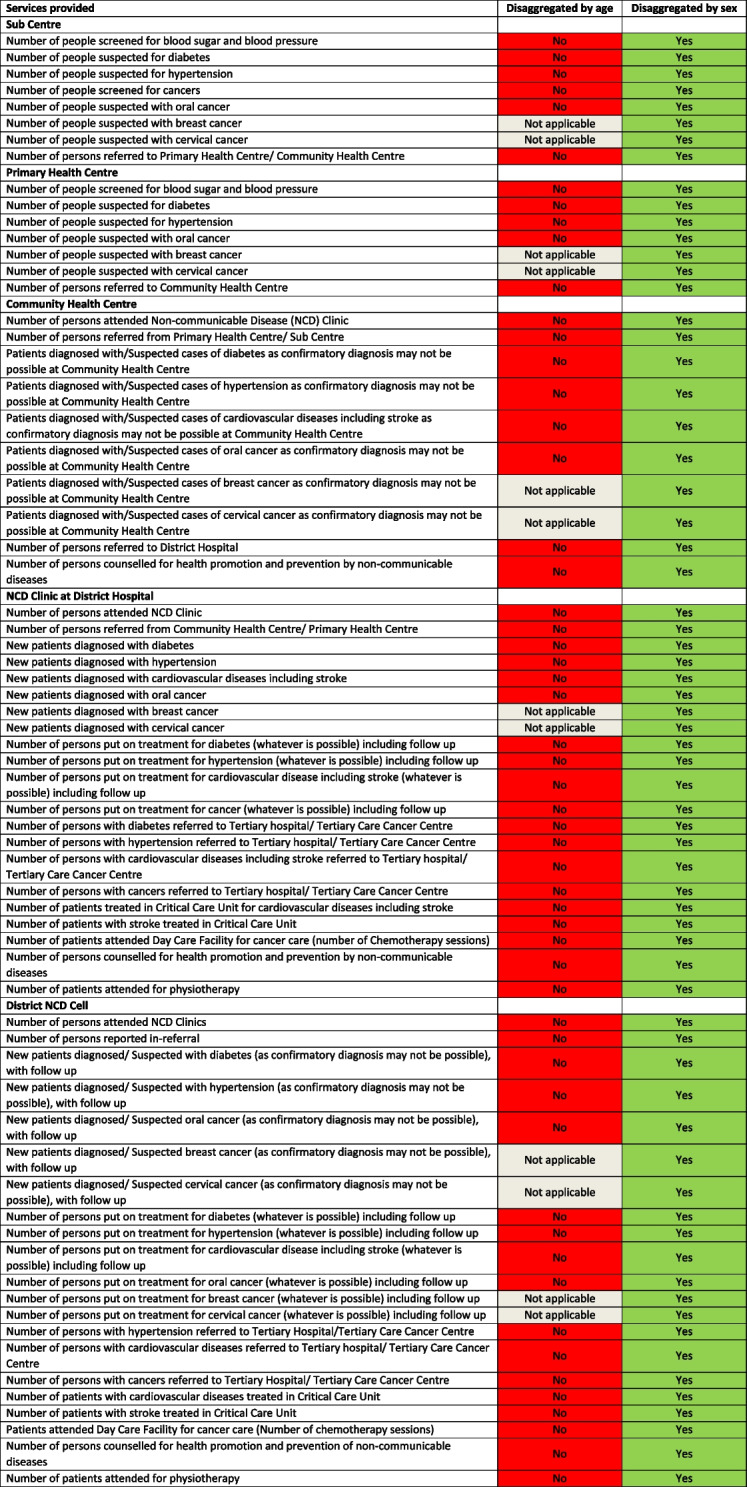
Age- and sex- disaggregation in the available indicators for monitoring services for older persons in the National Program for the Prevention and Control of Non-Communicable Diseases (NP-NCD)

## Discussion

The older persons accounted for nearly one-third of the total disease burden in India, while accounting for only 10% of the total population in 2019 in India. Most of the disease burden in the older persons were accounted for by mortality, with the burden of CMNNDs higher in females and that of NCDs and injuries higher in males. State-variations in the disease burden were relatively higher for CMNNDs and injuries as compared with NCDs. Importantly, the data capture of service utilisation indicators was not age- and sex-disaggregated for the most part to effectively monitor utilisation of health services by the older persons.

Broadly, the pattern of disease burden was similar for older females and males with NCDs as the leading cause of premature death and disability across all the older age groups and most states. Cardiovascular diseases, chronic respiratory diseases, neoplasms, diabetes, kidney diseases, and musculoskeletal disorders accounted for nearly 80% of DALYs from NCDs. Detailed analysis documenting the trends in burden of these diseases at the sub-national level from 1990 onwards have been published for all ages, highlighting increase in prevalence of these disease conditions and of their risk factors over time [17–22]. The gaps in data availability on morbidity and risk factors at the population-level for many of these diseases has been previously highlighted to improve the robustness of the DALY estimates, and to inform planning and efficient delivery of services [17–22]. In addition, given that although primary prevention in adults aged ≤ 60 years can improve health in successive cohorts of older people, there is much more potential to reduce disease burden from more effective primary, secondary, and tertiary prevention targeting the older persons.

Notably, there are variations in the magnitude of disease burden by specific diseases and conditions not only between older females and males, but also by age groups within females and males particularly for CMNNDs and injuries. The pattern for diarrhoeal diseases and tuberculosis, and for falls and road injuries varied considerably by age in females and males under CMNNDs and injuries, respectively. Under NCDs, the pattern for chronic respiratory diseases and musculoskeletal disorders also showed notable age-related variation, with a more pronounced difference in females than in males. As with the significant variations in the overall pattern and magnitude of disease burden between the states, [11] this burden in the older persons also varied greatly between females and males, and within females and males at the state-level. This comprehensive mapping of inequalities in disease burden across the states of India for the older persons can be a crucial input for more specific health service planning for the older persons in each state.

Injuries have not received much attention in the national programs despite the disease burden, which can have serious implications for improving older person’s health in India. Injuries accounted for 10–15% of YLLs across the various age categories in older females and males, with falls and road traffic injuries accounting for the majority of this burden. Injuries resulting from falls in older persons are a major public-health concern globally having negative effects on functional independence and quality of life and are associated with increased morbidity, disability, mortality and health-related costs [23]. A recent systematic review and meta-analysis reported falls in nearly one-third of the older persons in India, and highlighted the need to address methodological variations and quality of the data to design programs for prevention of falls and for rehabilitation [24]. With regard to road traffic crashes, a variety of actions are recommended to reduce road traffic mortality in India as it’s contribution to the global number of deaths due to road injuries has increased over time [25]. Specifically for the older persons, detailed understanding of their skills, risk factors, and roadway design will be needed to implement relevant actions to address the road traffic crash disease burden in them [26–29].

The Government of India has incorporated older person’s health in universal health coverage under the Ayushman Bharat program, thereby, showing a commitment to maintain health and function across the entire life course for Indian population [7]. Notably, India is also a member country in the Global Commission on Ageing in Developing Countries which was launched in 2013 to enable gender, equity and rights based policies and programming that will improve the quality of life of the ageing population in developing countries [30]. In addition to India being part of the WHO’s multi-country Study on global AGEing and adult health (SAGE), [31] launch of the Longitudinal Ageing Study in India (LASI) provides internationally comparable survey data on a variety of topics necessary for understanding the economic, social, psychological, and health aspects of adults and the ageing process [32]. Furthermore, the National Sample Surveys (NSS) provide data on economic independence and state of health for the older persons [33–36]. In addition to trends in prevalence of diseases, national data collection efforts such as LASI and NSS not only facilitate monitoring of trends in healthy life expectancies for the older persons, [37, 38] but such data can enable health interventions that focus on improving the functioning of older persons within an integrated people-centred care strategy across the entire continuum of care [39].

The commitment towards older person’s health will need concerted evidence generation that can be converted into practice. Studies such as SAGE, LASI and NSS will need to be complemented by assessments of effectiveness of the existing health financing, human resource, essential medicines or technology, and service delivery approaches targeted at the older persons [40]. Post-graduate training opportunities in geriatrics are currently limited in India, [41, 42] which will need significant expansion to cater to the growing disease burden in the older persons in the country. Given the selective utilisation of health services, higher in-patient spending among the older persons, and role of private and social health insurance to reducing these costs, thrust on insurance-financed health systems to reduce health spending among the older persons is also recommended, for which a disease-specific policy is required for the older persons along with ensuring state-of-the-art treatment facilities for them in public hospitals for critical ailments [43]. Essential actions to improve health of the older persons, with NCDs in particular, will need to extend beyond a focus on prevention of early mortality to include provision of chronic care for key non-fatal disorders that affect the function of older populations [40]. Despite accounting for significant morbidity among the older persons, and with the burden projected to increase, [44] musculoskeletal disorders have not received much attention in the public health programs as compared to diseases causing high mortality. [45] Strengthening health systems to improve prevention and management of musculoskeletal disorders is crucial [46]. While the National Ayush Mission has adopted an integrated approach for the management of osteoarthritis and other musculoskeletal disorders, [15] there is a need to align this with the broader NCD initiatives and prioritise gender-sensitive interventions [44, 45, 47, 48].

Increased longevity is accompanied by complexity and multi-morbidity or multiple long-term conditions (two or more chronic conditions), [49–58] however, GBD estimates currently do not account for multi-morbidity. Significant gaps continue to exist in data availability that enables an understanding of the burden, management, and outcomes of patients with multimorbidity. Furthermore, clinical guidelines for chronic illnesses as well as most health systems almost always focus on one disorder, although most people with those disorders will have multimorbidity, [59] which leads to questions about whether treatments and services that are developed in otherwise healthy people work in people with many health problems. Given that individuals with multimorbidity are far more susceptible to significant declines in both physical and mental health, resulting in increased disability, decreased quality of life, higher mortality and increased healthcare utilisation and costs, compared to those with single morbidity, it is imperative that researchers, healthcare providers and policymakers accord adequate recognition and address it appropriately. Thus, health systems need to reorient their focus from individual disease management to having an integrated person-centred care model. This should be complemented with better research using standardised definitions and outcome measures to understand all facets of multimorbidity, from disease mechanisms through to treatment, that are needed for the older persons, reflecting the health needs of the population driving the future trends [55].

The findings presented in this paper highlight that the disease burden in the older population is not homogeneous, and age- and sex-disaggregated disease burden understanding is imperative for the programs and policies to effectively respond to their wellness needs. Age- and sex-disaggregated service utilisation data capture on a routine basis across the national programs that cater to the older persons would facilitate not only planning of services for them but help identify those who may be unable to access these services. In the light of the recent global evidence on impact of COVID-19 pandemic on the older persons, such data can highlight vulnerability of the older persons to put corrective actions in place to mitigate their vulnerability [60]. Focus on sex-disaggregated disease burden for the older persons is extremely important because feminisation of gender disparities exist at all ages but when women become old, the consequences become more acute. Incidence of widowhood and higher life expectancy among older women are key demographic characteristics in India [3]. Poverty is inherently gendered in old age when older women are more likely to be widowed, living alone, with no income and with fewer assets of their own, and fully dependent on family for support [3]. With the progressive increase in the number of older women compared to older men with advancing age, it will be important for the policies and programes to focus on the special needs of older women.

There are some limitations to the analysis presented. The review of national program service utilisation indicators undertaken within the context of disease burden is based on the documentation for these programs available in the public domain, and not based on the actual implementation of the programs on the ground such as the availability and utilisation of services under these programs was beyond the scope of the analysis undertaken. The general limitations of GBD methods are published elsewhere [8–11]. A specific limitation for India is an incomplete medically certified cause of death system that covers only a small proportion of the deaths in India and has variable coverage across the states [61]. Additionally, the COVID-19 pandemic has caused huge health, social and economic disruption to the older persons, [60, 61] however, that assessment is beyond the scope of this paper. The strengths of the analysis presented are the comparable disease burden as estimated by the GBD Study across age groups for the older persons, by sex and by state, and the availability of this disease burden considering fatal and non-fatal health outcomes that allows for more in-depth interpretation of the disease burden focus within the national health programs addressing the health of older persons in India. The review of service utilisation indicators in these programs within the context of disease burden, and the gaps highlighted in the availability of service indicators are major strengths of the analysis presented.

## Conclusions

In conclusion, the analysis presented in this paper provides a much-needed synthesis of the evidence on disease burden among the older persons across age groups and by sex, to facilitate further improvements in the health and wellbeing of the older persons in India.

## Supplementary Information

Supplementary Material 1.

## Data Availability

The data used for estimating disease burden in this paper are available at http://ghdx.healthdata.org/gbd-2019, https://vizhub.healthdata.org/gbd-compare/india, and from the authors on request.

## Abbreviations

CMNNDs: Communicable, maternal, neonatal, and nutritional diseases
DALYs: Disability-adjusted life years
GBD: Global Burden of Diseases, Injuries, and Risk Factors Study
HWCs: Health and Wellness Centres
LASI: Longitudinal Ageing Study in India
MSDP: National Program for Prevention and Management of Osteoarthritis and Musculoskeletal DisordersProgram
NSS: National Sample Survey
NCDs: Non-communicable diseases
NMPH: National Mental Health Program
NPCBVI: National Program for Control of Blindness and Visual Impairment
NPHCE: National Program for Health Care of the Elderly
NP-NCD: National Program for Prevention and Control of NCDs
NPPCD: National Program for Prevention and Control of Deafness
UIs: Uncertainty intervals
WHO: World Health Organization
YLDs: Years lived with disability
YLLs: Years of life lost
