# Supplementary material for: Age- and sex-disaggregated disease burden among the older persons in India
Source: BMC Geriatr. 2024 Dec 19;24:1019. doi: 10.1186/s12877-024-05614-w (PMC11661056; doi:10.1186/s12877-024-05614-w)
Supplement: Supplementary file 1 — Supplementary Material 1. [file 12877_2024_5614_MOESM1_ESM.docx]

**Revision**

**26 October 2024**

**Age- and sex-disaggregated disease burden among the older persons in India**

**Supplementary File**

Correspondence to: Prof. Rakhi Dandona, [rakhi.dandona@phfi.org](mailto:rakhi.dandona@phfi.org)

**Table of contents**

[Supplementary Table 1. Disability-adjusted life years (DALYs) and years of life lost (YLLs) as a percent of DALYs by age group for major disease groups for the population aged 60 years or more in India in 2019, the Global Burden of Disease Study. 3](#_Toc185237656)

[Supplementary Table 2. Rates of disability-adjusted life years (DALYs), years of life lost (YLLs) and years lived with disability (YLDs) by age group for major disease groups for the population aged 60 years or more in India in 2019, the Global Burden of Disease Study. 4](#_Toc185237657)

[Supplementary Table 3. Top 10 causes of years of life lost (YLLs) and of years lived with disability (YLDs) among females 60 years or more in India in 2019, the Global Burden of Disease Study. 5](#_Toc185237658)

[Supplementary Table 4. Top 10 causes of years of life lost (YLLs) and of years lived with disability (YLDs) among males 60 years or more in India in 2019, the Global Burden of Disease Study. 6](#_Toc185237659)

[Supplementary Figure 1. Percent contribution of level 3 diseases to the total disability-adjusted life years (DALYs) due to communicable, maternal, neonatal, and nutritional diseases (CMNNDs) in the population aged 60 years or more by sex in India in 2019, the Global Burden of Disease Study. 7](#_Toc185237660)

[Supplementary Figure 2. Percent contribution of level 3 diseases to the total disability-adjusted life years (DALYs) due to non-communicable diseases (NCDs) in the population aged 60 years or more by sex in India in 2019, the Global Burden of Disease Study. 8](#_Toc185237661)

[Supplementary Figure 3. Percent contribution of level 3 diseases to the total disability-adjusted life years (DALYs) due to injuries in the population aged 60 years or more by sex in India in 2019, the Global Burden of Disease Study. 9](#_Toc185237662)

[Supplementary Figure 4. Percent contribution of years of life lost (YLLs) and years lived with disability (YLDs) to the total disability-adjusted life years (DALYs) among the population aged 60 years or more by sex in India in 2019, the Global Burden of Disease Study. 10](#_Toc185237663)

[Supplementary Table 5. Crude DALY rate of communicable, maternal, neonatal, and nutritional diseases (CMNNDs), non-communicable diseases (NCDs) and injuries in the population aged 60 years or more by sex in the states of India in 2019, the Global Burden of Disease Study. EAG refers to Empowered Action Group 11](#_Toc185237664)

[Supplementary Table 6. Age- and sex- disaggregation in the available indicators for services provided to the older persons aged 60 years or more under other relevant national health programs. 12](#_Toc185237665)

[Supplementary Table 7. Age- and sex- disaggregation in the available indicators for monitoring services for older persons aged 60 years or more at the Health and Wellness Centres (HWCs). 14](#_Toc185237666)

# **Supplementary Table 1. Disability-adjusted life years (DALYs) and years of life lost (YLLs) as a percent of DALYs by age group for major disease groups for the population aged 60 years or more in India in 2019, the Global Burden of Disease Study.**

| **Age group** | **Females** | | **Males** | | **Both sexes combined** | |
| --- | --- | --- | --- | --- | --- | --- |
|  | **DALYs (in thousands)**  **(95% uncertainty interval)** | **YLLs as a percent of DALYs** | **DALYs (in thousands)**  **(95% uncertainty interval)** | **YLLs as a percent of DALYs** | **DALYs (in thousands)**  **(95% uncertainty interval)** | **YLLs as a percent of DALYs** |
| **60-64 years** | | | | | | |
| CMNNDs | 2,146 (1,644 to 2,883) | 72.6 | 2,085 (1,685 to 2,662) | 80.2 | 4,231 (3,499 to 5,162) | 76.3 |
| NCDs | 11,909 (10,034 to 13,765) | 63.1 | 13,376 (11,083 to 15,841) | 72.8 | 25,285 (22,110 to 28,829) | 68.2 |
| Injuries | 1,099 (902 to 1,305) | 57.5 | 1,363 (1,108 to 1,650) | 58.6 | 2,462 (2,062 to 2,897) | 58.1 |
| Total | 15,153 (12,858 to 17,462) | 64.1 | 16,825 (14,013 to 19,992) | 72.5 | 31,978 (28,047 to 36,468) | 68.5 |
| **65-69 years** | | | | | | |
| CMNNDs | 2,308 (1,732 to 3,199) | 76.9 | 2,059 (1,658 to 2,750) | 82.8 | 4,367 (3,584 to 5,442) | 79.7 |
| NCDs | 11,891 (10,095 to 13,643) | 66.1 | 13,421 (11,213 to 15,847) | 74.4 | 25,312 (22,381 to 28,612) | 70.5 |
| Injuries | 1,105 (900 to 1,308) | 58.7 | 1,246 (1,010 to 1,494) | 60.2 | 2,350 (1,955 to 2,741) | 59.5 |
| Total | 15,304 (13,175 to 17,427) | 67.2 | 16,726 (14,050 to 19,628) | 74.3 | 32,030 (28,364 to 36,061) | 70.9 |
| **70-74 years** | | | | | | |
| CMNNDs | 2,250 (1,637 to 3,240) | 82.7 | 1,911 (1,529 to 2,592) | 83.5 | 4,161 (3,365 to 5,354) | 83.1 |
| NCDs | 10,173 (8,677 to 11,592) | 69.3 | 11,298 (9,530 to 13,182) | 75.7 | 21,470 (19,135 to 24,046) | 72.7 |
| Injuries | 951 (778 to 1,122) | 59.2 | 951 (776 to 1,131) | 60.5 | 1,902 (1,617 to 2,224) | 59.9 |
| Total | 13,373 (11,582 to 15,166) | 70.9 | 14,160 (12,040 to 16,508) | 75.7 | 27,533 (24,529 to 30,719) | 73.3 |
| **75-79 years** | | | | | | |
| CMNNDs | 1,802 (1,253 to 2,639) | 84.4 | 1,437 (1,154 to 1,985) | 84.7 | 3,239 (2,577 to 4,196) | 84.6 |
| NCDs | 7,984 (6,771 to 9,093) | 71.3 | 8,201 (6,915 to 9,452) | 76.9 | 16,185 (14,421 to 18,043) | 74.2 |
| Injuries | 793 (653 to 945) | 58.6 | 689 (563 to 810) | 62.2 | 1,483 (1,254 to 1,731) | 60.3 |
| Total | 10,579 (9,197 to 11,951) | 72.6 | 10,327 (8,891 to 11,870) | 77.0 | 20,906 (18,903 to 23,154) | 74.8 |
| **80 years and more** | | | | | | |
| CMNNDs | 2,523 (1,661 to 3,847) | 91.1 | 1,625 (1,281 to 2,315) | 89.4 | 4,149 (3,154 to 5,575) | 90.4 |
| NCDs | 9,608 (8,073 to 10,937) | 75.1 | 8,167 (7,034 to 9,195) | 78.7 | 17,775 (15,778 to 19,642) | 76.8 |
| Injuries | 1,010 (830 to 1,186) | 58.9 | 687 (573 to 803) | 64.2 | 1,697 (1,429 to 1,955) | 61.1 |
| Total | 13,142 (11,679 to 14,535) | 76.9 | 10,479 (9,268 to 11,729) | 79.4 | 23,620 (21,677 to 25,618) | 78.0 |
| **60 years and more** | | | | | | |
| CMNNDs | 11,028 (8,010 to 15,576) | 81.7 | 9,117 (7,401 to 12,237) | 83.8 | 20,146 (16,380 to 25,498) | 82.7 |
| NCDs | 51,564 (44,013 to 58,694) | 68.5 | 54,463 (45,794 to 63,476) | 75.3 | 1,06,027 (94,348 to 1,19,053) | 72.0 |
| Injuries | 4,958 (4,081 to 5,827) | 58.6 | 4,936 (4,033 to 5,863) | 60.6 | 9,894 (8,428 to 11,509) | 59.6 |
| Total | 67,551 (58,575 to 76,596) | 70.0 | 68,516 (58,277 to 79,716) | 75.4 | 1,36,067 (1,21,340 to 1,51,876) | 72.7 |
| **All ages** | | | | | | |
| Total | 2,27,393 (1,97,334 to 2,60,714) |  | 2,40,482 (2,09,055 to 2,75,980) |  | 4,67,876 (4,15,925 to 5,26,872) |  |

CMNNDs= Communicable, maternal, neonatal, and nutritional diseases; NCDs= Non-communicable diseases

# **Supplementary Table 2. Rates of disability-adjusted life years (DALYs), years of life lost (YLLs) and years lived with disability (YLDs) by age group for major disease groups for the population aged 60 years or more in India in 2019, the Global Burden of Disease Study.**

| **Age group** | **Females** | | | **Males** | | |
| --- | --- | --- | --- | --- | --- | --- |
|  | **DALY rate per 100,000 population**  **(95% uncertainty interval)** | **YLL rate per 100,000 population**  **(95% uncertainty interval)** | **YLD rate per 100,000 population**  **(95% uncertainty interval)** | **DALY rate per 100,000 population**  **(95% uncertainty interval)** | **YLL rate per 100,000 population**  **(95% uncertainty interval)** | **YLD rate per 100,000 population**  **(95% uncertainty interval)** |
| **60-64 years** | | | | | | |
| CMNNDs | 9,390 (7,193-12,616) | 6,813 (4,753-10,107) | 2,577 (1,871-3,424) | 9,431 (7,620-12,042) | 7,568 (5,812-10,092) | 1,863 (1,376-2,504) |
| NCDs | 52,118 (43,914-60,241) | 32,903 (25,983-39,691) | 19,214 (14,515-24,675) | 60,503 (50,133-71,653) | 44,027 (35,339-54,114) | 16,476 (12,453-21,350) |
| Injuries | 4,810 (3,947-5,710) | 2,765 (2,076-3,483) | 2,045 (1,501-2,667) | 6,166 (5,014-7,462) | 3,612 (2,735-4,515) | 2,554 (1,857-3,375) |
| Total | 66,317 (56,270-76,422) | 42,481 (34,946-50,998) | 23,836 (17,959-30,546) | 76,101 (63,384-90,429) | 55,207 (44,452-67,557) | 20,894 (15,704-26,941) |
| **65-69 years** | | | | | | |
| CMNNDs | 12,328 (9,252-17,085) | 9,484 (6,639-14,250) | 2,844 (2,076-3,785) | 11,488 (9,250-15,341) | 9,510 (7,314-13,324) | 1,979 (1,431-2,634) |
| NCDs | 63,502 (53,910-72,863) | 42,000 (33,880-49,796) | 21,503 (16,437-27,019) | 74,886 (62,566-88,419) | 55,679 (45,125-67,424) | 19,206 (14,675-24,199) |
| Injuries | 5,901 (4,807-6,985) | 3,462 (2,584-4,294) | 2,438 (1,790-3,192) | 6,949 (5,635-8,334) | 4,184 (3,146-5,221) | 2,765 (2,022-3,648) |
| Total | 81,731 (70,360-93,069) | 54,946 (46,198-64,640) | 26,785 (20,282-34,057) | 93,323 (78,393-1,09,514) | 69,373 (56,596-83,808) | 23,950 (18,162-30,495) |
| **70-74 years** | | | | | | |
| CMNNDs | 17,165 (12,491-24,719) | 14,198 (9,717-21,989) | 2,967 (2,181-3,891) | 15,313 (12,255-20,767) | 12,781 (9,868-18,128) | 2,532 (1,828-3,342) |
| NCDs | 77,621 (66,212-88,448) | 53,815 (43,305-63,489) | 23,806 (18,291-29,597) | 90,528 (76,363-1,05,629) | 68,489 (56,209-81,945) | 22,039 (16,824-27,532) |
| Injuries | 7,255 (5,938-8,564) | 4,292 (3,191-5,318) | 2,962 (2,211-3,878) | 7,621 (6,215-9,060) | 4,614 (3,516-5,624) | 3,008 (2,210-3,939) |
| Total | 102,040 (88,374-115,726) | 72,305 (61,428-84,233) | 29,736 (22,839-37,323) | 113,462 (96,476-132,273) | 85,884 (70,896-102,560) | 27,578 (21,133-34,846) |
| **75-79 years** | | | | | | |
| CMNNDs | 20,576 (14,313-30,135) | 17,375 (11,467-27,312) | 3,201 (2,358-4,154) | 18,652 (14,972-25,767) | 15,801 (12,265-22,645) | 2,851 (2,086-3,776) |
| NCDs | 91,183 (77,330-103,848) | 65,050 (52,554-76,451) | 26,134 (20,104-32,347) | 106,439 (89,752-122,673) | 81,851 (68,175-97,342) | 24,588 (18,936-30,637) |
| Injuries | 9,062 (7,458-10,788) | 5,311 (3,992-6,513) | 3,751 (2,778-5,059) | 8,944 (7,312-10,517) | 5,560 (4,298-6,773) | 3,384 (2,481-4,477) |
| Total | 120,821 (105,045-136,492) | 87,735 (75,253-101,296) | 33,086 (25,471-41,530) | 134,035 (115,395-154,068) | 103,212 (86,727-121,038) | 30,822 (23,573-38,960) |
| **80 years and more** | | | | | | |
| CMNNDs | 30,786 (20,267-46,929) | 28,034 (17,596-44,115) | 2,752 (2,043-3,555) | 25,901 (20,421-36,901) | 23,159 (17,724-34,036) | 2,742 (2,015-3,574) |
| NCDs | 117,223 (98,488-133,440) | 88,021 (70,083-1,02,628) | 29,202 (22,601-35,829) | 130,158 (112,105-146,549) | 1,02,496 (86,710-1,17,432) | 27,662 (21,204-34,232) |
| Injuries | 12,325 (10,130-14,472) | 7,265 (5,427-8,941) | 5,059 (3,741-6,673) | 10,944 (9,132-12,800) | 7,025 (5,555-8,225) | 3,918 (2,857-5,185) |
| Total | 160,334 (142,491-177,331) | 123,321 (108,693-138,764) | 37,013 (28,556-45,992) | 167,003 (147,701-186,927) | 132,681 (115,987-149,837) | 34,322 (26,233-42,841) |
| **60 years and more** | | | | | | |
| CMNNDs | 15,396 (11,183-21,744) | 12,581 (8,570-18,963) | 2,814 (2,065-3,711) | 13,712 (11,131-18,404) | 11,495 (8,981-16,086) | 2,217 (1,634-2,906) |
| NCDs | 71,984 (61,444-81,935) | 49,343 (39,744-58,003) | 22,641 (17,376-28,354) | 81,912 (68,874-95,456) | 61,660 (50,754-73,886) | 20,252 (15,519-25,447) |
| Injuries | 6,922 (5,698-8,134) | 4,053 (3,118-4,991) | 2,869 (2,137-3,762) | 7,423 (6,065-8,817) | 4,502 (3,491-5,477) | 2,921 (2,143-3,842) |
| Total | 94,302 (81,774-106,922) | 65,977 (56,102-76,829) | 28,325 (21,606-35,797) | 103,047 (87,652-119,886) | 77,657 (64,343-92,469) | 25,390 (19,343-32,309) |

CMNNDs= Communicable, maternal, neonatal, and nutritional diseases; NCDs= Non-communicable diseases

# **Supplementary Table 3. Top 10 causes of years of life lost (YLLs) and of years lived with disability (YLDs) among females 60 years or more in India in 2019, the Global Burden of Disease Study.**

| **60-64 years** | | **65-69 years** | | **70-74 years** | | **75-79 years** | | **80+ years** | |
| --- | --- | --- | --- | --- | --- | --- | --- | --- | --- |
| **Top 10 individual causes of YLLs** | **Percent of total YLLs (95% UIs)** | **Top 10 individual causes of YLLs** | **Percent of total YLLs (95% UIs)** | **Top 10 individual causes of YLLs** | **Percent of total YLLs (95% UIs)** | **Top 10 individual causes of YLLs** | **Percent of total YLLs (95% UIs)** | **Top 10 individual causes of YLLs** | **Percent of total YLLs (95% UIs)** |
| Ischemic heart disease | 17.6 (15.5-19.4) | Ischemic heart disease | 17.5 (15.4-19.3) | Ischemic heart disease | 17.1 (14.5-19.0) | Ischemic heart disease | 17.3 (14.4-19.5) | Ischemic heart disease | 16.4 (13.1-18.9) |
| Stroke | 11.2 (9.8-12.9) | Stroke | 11.5 (10.0-13.0) | Chronic obstructive pulmonary disease | 13.3 (9.5-16.1) | Chronic obstructive pulmonary disease | 14.1 (9.8-17.1) | Chronic obstructive pulmonary disease | 16.0 (11.0-19.8) |
| Chronic obstructive pulmonary disease | 8.6 (5.9-10.6) | Chronic obstructive pulmonary disease | 10.5 (7.6-12.7) | Stroke | 10.3 (8.8-12.0) | Diarrheal diseases | 10.9 (3.8-22.9) | Diarrheal diseases | 14.5 (5.8-28.4) |
| Diarrheal diseases | 6.4 (2.0-14.1) | Diarrheal diseases | 7.5 (2.5-16.5) | Diarrheal diseases | 9.9 (3.4-21.1) | Stroke | 10.2 (8.5-11.8) | Stroke | 7.8 (6.2-9.4) |
| Diabetes mellitus | 4.7 (4.1-5.3) | Lower respiratory infections | 4.6 (3.7-5.4) | Lower respiratory infections | 5.1 (4.1-6.2) | Lower respiratory infections | 4.8 (3.9-5.9) | Lower respiratory infections | 4.9 (4.0-6.0) |
| Tuberculosis | 3.9 (3.0-4.9) | Diabetes mellitus | 4.3 (3.7-4.9) | Diabetes mellitus | 3.6 (3.1-4.2) | Diabetes mellitus | 3.9 (3.4-4.5) | Alzheimer's disease and other dementias | 4.7 (1.1-13.0) |
| Lower respiratory infections | 3.7 (2.9-4.4) | Tuberculosis | 3.3 (2.6-4.3) | Asthma | 3.4 (1.9-5.3) | Falls | 3.8 (2.8-4.6) | Falls | 4.3 (3.2-5.2) |
| Asthma | 3.2 (1.9-4.8) | Asthma | 3.2 (1.8-4.8) | Falls | 3.4 (2.4-4.0) | Asthma | 3.3 (1.7-5.0) | Diabetes mellitus | 3.4 (2.8-4.0) |
| Chronic kidney disease | 3.2 (2.8-3.5) | Falls | 2.9 (1.9-3.5) | Tuberculosis | 3.0 (2.3-3.9) | Tuberculosis | 2.6 (2.0-3.4) | Asthma | 3.3 (1.8-4.9) |
| Breast cancer | 2.8 (2.4-3.2) | Chronic kidney disease | 2.6 (2.3-2.9) | Chronic kidney disease | 2.2 (1.9-2.5) | Hypertensive heart disease | 2.4 (1.4-3.4) | Hypertensive heart disease | 2.6 (1.6-3.8) |
| **Top 10 individual causes of YLDs** | **Percent of total YLDs (95% UIs)** | **Top 10 individual causes of YLDs** | **Percent of total YLDs (95% UIs)** | **Top 10 individual causes of YLDs** | **Percent of total YLDs (95% UIs)** | **Top 10 individual causes of YLDs** | **Percent of total YLDs (95% UIs)** | **Top 10 individual causes of YLDs** | **Percent of total YLDs (95% UIs)** |
| Other musculoskeletal disorders* | 7.9 (5.4-11.0) | Blindness and vision loss | 9.1 (7.6-11.0) | Blindness and vision loss | 10.4 (8.8-12.3) | Blindness and vision loss | 11.1 (9.6-12.8) | Blindness and vision loss | 12.0 (10.5-13.6) |
| Low back pain | 7.8 (5.6-10.6) | Diabetes mellitus | 7.8 (6.6-9.1) | Chronic obstructive pulmonary disease | 8.0 (6.0-10.4) | Chronic obstructive pulmonary disease | 9.3 (6.9-12.1) | Chronic obstructive pulmonary disease | 11.1 (8.5-14.5) |
| Diabetes mellitus | 7.6 (6.4-8.9) | Low back pain | 6.9 (4.8-9.5) | Diabetes mellitus | 7.5 (6.3-8.9) | Age-related and other hearing loss | 8.1 (6.5-9.8) | Falls | 10.2 (8.7-11.8) |
| Blindness and vision loss | 7.6 (6.0-9.5) | Other musculoskeletal disorders* | 6.7 (4.7-9.5) | Age-related and other hearing loss | 7.2 (5.7-8.9) | Falls | 7.3 (6.3-8.8) | Age-related and other hearing loss | 9.3 (7.9-10.8) |
| Depressive disorders | 5.9 (4.3-7.7) | Age-related and other hearing loss | 6.4 (4.9-8.2) | Low back pain | 6.2 (4.3-8.5) | Diabetes mellitus | 6.9 (6.0-8.1) | Diabetes mellitus | 6.2 (5.3-7.2) |
| Dietary iron deficiency | 5.8 (4.8-6.7) | Chronic obstructive pulmonary disease | 6.4 (4.7-8.4) | Other musculoskeletal disorders* | 5.5 (3.5-8.2) | Low back pain | 6.0 (4.3-8.4) | Low back pain | 5.8 (4.4-7.7) |
| Age-related and other hearing loss | 5.7 (4.3-7.4) | Dietary iron deficiency | 6.0 (5.0-6.9) | Falls | 5.4 (4.6-6.3) | Dietary iron deficiency | 5.0 (4.2-5.8) | Alzheimer's disease and other dementias | 3.8 (2.8-4.9) |
| Chronic obstructive pulmonary disease | 4.8 (3.6-6.2) | Depressive disorders | 5.1 (3.8-6.5) | Dietary iron deficiency | 5.3 (4.4-6.1) | Other musculoskeletal disorders* | 3.9 (2.5-5.7) | Dietary iron deficiency | 3.3 (2.7-3.9) |
| Road injuries | 3.6 (3.2-4.0) | Falls | 4.1 (3.5-4.7) | Depressive disorders | 4.3 (3.2-5.6) | Depressive disorders | 3.7 (2.6-4.9) | Osteoarthritis | 2.9 (1.7-5.4) |
| Osteoarthritis | 3.4 (2.0-6.3) | Osteoarthritis | 3.4 (2.0-6.4) | Osteoarthritis | 3.4 (2.0-6.4) | Osteoarthritis | 3.2 (1.9-6.0) | Depressive disorders | 2.9 (2.1-3.9) |

*Other musculoskeletal disorders include lupus erythematosus, infectious arthropathies, inflammatory polyarthropathies, other joint disorders, systemic connective tissue disorders, deforming dorsopathies, spondylopathies, disorders of muscles, disorders of

synovium and tendon, other soft tissue disorders, disorders of bone density and structure, osteomyelitis, other osteopathies, chondropathies, and other disorders of the MSK system and connective tissue.

UIs=Uncertainty intervals

|  | Communicable, maternal, neonatal, and nutritional diseases |
| --- | --- |
|  | Non-communicable diseases |
|  | Injuries |

# **Supplementary Table 4. Top 10 causes of years of life lost (YLLs) and of years lived with disability (YLDs) among males 60 years or more in India in 2019, the Global Burden of Disease Study.**

| **60-64 years** | | **65-69 years** | | **70-74 years** | | **75-79 years** | | **80+ years** | |
| --- | --- | --- | --- | --- | --- | --- | --- | --- | --- |
| **Top 10 individual causes of YLLs** | **Percent of total YLLs (95% UIs)** | **Top 10 individual causes of YLLs** | **Percent of total YLLs (95% UIs)** | **Top 10 individual causes of YLLs** | **Percent of total YLLs (95% UIs)** | **Top 10 individual causes of YLLs** | **Percent of total YLLs (95% UIs)** | **Top 10 individual causes of YLLs** | **Percent of total YLLs (95% UIs)** |
| Ischemic heart disease | 23.7 (22.3-25.1) | Ischemic heart disease | 21.9 (20.5-23.4) | Ischemic heart disease | 20.9 (19.4-22.3) | Ischemic heart disease | 20.3 (18.6-21.8) | Ischemic heart disease | 20.3 (18.0-21.9) |
| Chronic obstructive pulmonary disease | 10.4 (8.2-11.9) | Chronic obstructive pulmonary disease | 13.2 (10.6-15.0) | Chronic obstructive pulmonary disease | 15.9 (12.8-17.9) | Chronic obstructive pulmonary disease | 17.3 (13.9-19.6) | Chronic obstructive pulmonary disease | 18.0 (14.3-20.3) |
| Stroke | 10.0 (9.2-10.9) | Stroke | 10.8 (9.9-11.7) | Stroke | 10.2 (9.3-11.2) | Stroke | 9.9 (8.9-11.0) | Diarrheal diseases | 8.3 (5.0-17.0) |
| Tuberculosis | 6.6 (5.7-7.4) | Tuberculosis | 5.4 (4.7-6.1) | Diarrheal diseases | 5.1 (3.0-11.3) | Diarrheal diseases | 5.9 (3.4-12.3) | Stroke | 7.9 (6.8-9.0) |
| Cirrhosis and other chronic liver diseases | 4.3 (3.9-4.8) | Diarrheal diseases | 3.9 (2.3-8.5) | Tuberculosis | 4.8 (4.1-5.4) | Lower respiratory infections | 4.3 (3.6-5.1) | Lower respiratory infections | 4.9 (4.1-5.9) |
| Diabetes mellitus | 3.8 (3.4-4.2) | Diabetes mellitus | 3.8 (3.4-4.2) | Lower respiratory infections | 3.8 (3.2-4.4) | Diabetes mellitus | 4.0 (3.6-4.4) | Alzheimer's disease and other dementias | 4.1 (1.0-11.5) |
| Chronic kidney disease | 3.2 (2.9-3.6) | Cirrhosis and other chronic liver diseases | 3.2 (2.9-3.8) | Diabetes mellitus | 3.8 (3.4-4.2) | Tuberculosis | 3.9 (3.3-4.5) | Diabetes mellitus | 3.6 (3.2-4.0) |
| Diarrheal diseases | 3.0 (1.8-6.8) | Lower respiratory infections | 3.1 (2.7-3.6) | Chronic kidney disease | 2.9 (2.6-3.2) | Falls | 2.8 (2.2-3.4) | Falls | 3.2 (2.6-3.9) |
| Lower respiratory infections | 2.7 (2.3-3.1) | Chronic kidney disease | 3.1 (2.9-3.5) | Cirrhosis and other chronic liver diseases | 2.5 (2.2-3.0) | Chronic kidney disease | 2.5 (2.2-2.8) | Tuberculosis | 3.1 (2.7-3.6) |
| Asthma | 2.1 (1.3-3.1) | Asthma | 2.2 (1.3-3.3) | Falls | 2.4 (1.9-2.9) | Asthma | 2.2 (1.4-3.4) | Chronic kidney disease | 2.5 (2.1-2.8) |
| **Top 10 individual causes of YLDs** | **Percent of total YLDs (95% UIs)** | **Top 10 individual causes of YLDs** | **Percent of total YLDs (95% UIs)** | **Top 10 individual causes of YLDs** | **Percent of total YLDs (95% UIs)** | **Top 10 individual causes of YLDs** | **Percent of total YLDs (95% UIs)** | **Top 10 individual causes of YLDs** | **Percent of total YLDs (95% UIs)** |
| Diabetes mellitus | 10.1 (8.7-11.7) | Diabetes mellitus | 10.0 (8.5-11.7) | Blindness and vision loss | 10.0 (8.4-11.9) | Blindness and vision loss | 10.8 (9.3-12.5) | Blindness and vision loss | 12.1 (10.6-13.7) |
| Blindness and vision loss | 7.7 (6.2-9.7) | Blindness and vision loss | 9.1 (7.5-11.0) | Diabetes mellitus | 9.1 (7.8-10.7) | Chronic obstructive pulmonary disease | 9.6 (7.3-12.3) | Chronic obstructive pulmonary disease | 11.4 (8.9-14.4) |
| Road injuries | 7.0 (6.3-7.8) | Age-related and other hearing loss | 7.4 (5.6-9.3) | Chronic obstructive pulmonary disease | 8.4 (6.4-10.8) | Age-related and other hearing loss | 8.8 (7.2-10.6) | Age-related and other hearing loss | 10.0 (8.6-11.5) |
| Age-related and other hearing loss | 6.6 (4.9-8.6) | Chronic obstructive pulmonary disease | 7.0 (5.3-9.0) | Age-related and other hearing loss | 8.0 (6.4-9.8) | Diabetes mellitus | 8.2 (7.1-9.6) | Diabetes mellitus | 7.0 (6.0-8.2) |
| Other musculoskeletal disorders* | 5.9 (3.7-8.5) | Road injuries | 6.4 (5.8-7.1) | Road injuries | 5.7 (5.2-6.3) | Road injuries | 5.3 (4.8-5.8) | Falls | 5.1 (4.3-5.8) |
| Chronic obstructive pulmonary disease | 5.5 (4.2-7.0) | Other musculoskeletal disorders* | 4.9 (3.2-7.2) | Low back pain | 4.5 (3.1-6.2) | Low back pain | 4.5 (3.1-6.5) | Road injuries | 4.9 (4.4-5.5) |
| Depressive disorders | 4.9 (3.6-6.4) | Low back pain | 4.3 (2.9-6.0) | Other musculoskeletal disorders* | 3.8 (2.2-6.1) | Falls | 4.1 (3.6-4.8) | Alzheimer's disease and other dementias | 4.2 (3.1-5.5) |
| Low back pain | 4.2 (2.9-5.8) | Depressive disorders | 4.2 (3.2-5.4) | Dietary iron deficiency | 3.7 (3.1-4.4) | Dietary iron deficiency | 3.8 (3.1-4.6) | Low back pain | 4.0 (2.9-5.4) |
| Falls | 3.0 (2.6-3.4) | Falls | 3.2 (2.8-3.6) | Depressive disorders | 3.6 (2.6-4.7) | Depressive disorders | 3.1 (2.2-4.2) | Dietary iron deficiency | 3.1 (2.5-3.7) |
| Dietary iron deficiency | 2.9 (2.2-3.6) | Dietary iron deficiency | 2.9 (2.3-3.6) | Falls | 3.5 (3.0-3.9) | Osteoarthritis | 2.6 (1.6-5.0) | Oral disorders | 2.8 (2.0-3.8) |

*Other musculoskeletal disorders include lupus erythematosus, infectious arthropathies, inflammatory polyarthropathies, other joint disorders, systemic connective tissue disorders, deforming dorsopathies, spondylopathies, disorders of muscles, disorders of

synovium and tendon, other soft tissue disorders, disorders of bone density and structure, osteomyelitis, other osteopathies, chondropathies, and other disorders of the MSK system and connective tissue.

UIs=Uncertainty intervals

|  | Communicable, maternal, neonatal, and nutritional diseases |
| --- | --- |
|  | Non-communicable diseases |
|  | Injuries |

# **Supplementary Figure 1. Percent contribution of level 3 diseases to the total disability-adjusted life years (DALYs) due to communicable, maternal, neonatal, and nutritional diseases (CMNNDs) in the population aged 60 years or more by sex in India in 2019, the Global Burden of Disease Study.**

Other CMNNDs include HIV/AIDS and sexually transmitted infections, enteric infections other than diarrheal diseases, neglected tropical diseases and malaria, nutritional deficiencies other than dietary iron deficiency, and other infectious diseases.

# **Supplementary Figure 2. Percent contribution of level 3 diseases to the total disability-adjusted life years (DALYs) due to non-communicable diseases (NCDs) in the population aged 60 years or more by sex in India in 2019, the Global Burden of Disease Study.**

Other NCDs include mental disorders, substance use disorders, skin and subcutaneous diseases, and other non-communicable diseases.

# **Supplementary Figure 3. Percent contribution of level 3 diseases to the total disability-adjusted life years (DALYs) due to injuries in the population aged 60 years or more by sex in India in 2019, the Global Burden of Disease Study.**

Other unintentional injuries include transport injuries other than road injuries, drowning, fire, heat and hot substances, poisonings, exposure to mechanical forces, adverse effects of medical treatment, animal contact, foreign body, environmental heat and cold exposure, exposure to forces of nature and all other unintentional injuries.

Other intentional injuries include interpersonal violence, conflict and terrorism, and executions and police conflict.

# **Supplementary Figure 4. Percent contribution of years of life lost (YLLs) and years lived with disability (YLDs) to the total disability-adjusted life years (DALYs) among the population aged 60 years or more by sex in India in 2019, the Global Burden of Disease Study.**

Communicable, maternal, neonatal, and nutritional diseases (CMNNDs)

Other CMNNDs include HIV/AIDS and sexually transmitted infections, enteric infections other than diarrheal diseases, neglected tropical diseases and malaria, nutritional deficiencies other than dietary iron deficiency, and other infectious diseases.

Non-communicable diseases (NCDs)

Other NCDs include mental disorders, substance use disorders, skin and subcutaneous diseases, and other non-communicable diseases.

Injuries

Other unintentional injuries include transport injuries other than road injuries, drowning, fire, heat and hot substances, poisonings, exposure to mechanical forces, adverse effects of medical treatment, animal contact, foreign body, environmental heat and cold exposure, exposure to forces of nature and all other unintentional injuries.

Other intentional injuries include interpersonal violence, conflict and terrorism, and executions and police conflict.

# **Supplementary Table 5. Crude DALY rate of communicable, maternal, neonatal, and nutritional diseases (CMNNDs), non-communicable diseases (NCDs) and injuries in the population aged 60 years or more by sex in the states of India in 2019, the Global Burden of Disease Study. EAG refers to Empowered Action Group**

| **States** | **DALY rate per 100,000 population (95% uncertainty intervals)** | | | | | |
| --- | --- | --- | --- | --- | --- | --- |
|  | **CMNNDs** | | **NCDs** | | **Injuries** | |
|  | **Females** | **Males** | **Females** | **Males** | **Females** | **Males** |
| **India** | **15,396 (11,183 to 21,745)** | **13,712 (11,130 to 18,405)** | **71,984 (61,442 to 81,937)** | **81,912 (68,874 to 95,468)** | **6,922 (5,697 to 8,135)** | **7,423 (6,065 to 8,818)** |
| **EAG states** |  |  |  |  |  |  |
| Assam | 16,920 (12,544 to 23,281) | 17,039 (13,381 to 21,670) | 78,985 (68,017 to 92,063) | 92,590 (76,301 to 1,10,940) | 5,216 (4,358 to 6,377) | 6,384 (5,157 to 8,012) |
| Bihar | 19,579 (12,770 to 31,411) | 16,501 (11,966 to 24,660) | 68,357 (55,574 to 80,902) | 73,278 (59,054 to 89,084) | 6,699 (5,331 to 8,135) | 6,843 (5,439 to 8,448) |
| Chhattisgarh | 22,994 (15,986 to 33,137) | 21,155 (16,429 to 28,045) | 76,130 (63,065 to 89,218) | 98,778 (82,385 to 1,13,167) | 7,847 (6,333 to 9,533) | 9,104 (7,513 to 10,714) |
| Jharkhand | 19,486 (13,852 to 27,222) | 14,458 (10,473 to 19,970) | 70,589 (59,910 to 80,681) | 55,818 (45,870 to 66,291) | 7,043 (5,845 to 8,219) | 6,207 (4,899 to 7,717) |
| Madhya Pradesh | 18,633 (13,188 to 28,008) | 17,918 (13,253 to 29,250) | 69,999 (58,535 to 79,945) | 87,497 (70,411 to 1,05,198) | 6,595 (5,525 to 7,798) | 7,702 (6,116 to 9,377) |
| Odisha | 21,381 (14,777 to 30,819) | 21,401 (15,365 to 29,337) | 64,894 (52,221 to 77,644) | 67,701 (55,347 to 83,581) | 7,443 (5,687 to 9,261) | 7,422 (5,981 to 9,245) |
| Rajasthan | 16,946 (11,779 to 25,840) | 16,511 (12,761 to 23,135) | 69,911 (56,996 to 82,686) | 94,471 (77,360 to 1,12,879) | 5,879 (4,753 to 7,212) | 7,330 (6,035 to 8,959) |
| Uttar Pradesh | 23,855 (16,802 to 35,283) | 21,959 (16,805 to 30,946) | 75,899 (62,380 to 88,719) | 99,242 (81,120 to 1,18,028) | 7,455 (5,954 to 9,032) | 8,833 (6,959 to 10,831) |
| Uttarakhand | 14,777 (11,606 to 19,350) | 16,725 (13,044 to 21,618) | 73,737 (63,301 to 83,662) | 1,04,972 (84,974 to 1,26,657) | 8,165 (6,707 to 9,714) | 9,433 (7,402 to 11,571) |
| **Other states** |  |  |  |  |  |  |
| Andhra Pradesh | 14,520 (9,954 to 22,596) | 11,298 (8,069 to 15,587) | 71,280 (58,414 to 85,445) | 73,993 (56,763 to 93,802) | 7,751 (6,061 to 9,582) | 7,341 (5,615 to 9,396) |
| Arunachal Pradesh | 14,035 (10,409 to 18,721) | 14,697 (10,515 to 19,321) | 69,990 (58,349 to 83,518) | 84,214 (67,241 to 1,01,142) | 6,051 (4,912 to 7,314) | 7,707 (6,144 to 9,635) |
| Delhi | 8,215 (6,370 to 10,693) | 7,667 (6,063 to 9,496) | 65,857 (57,108 to 75,089) | 72,412 (60,146 to 86,239) | 5,201 (4,297 to 6,100) | 5,725 (4,645 to 6,913) |
| Goa | 7,536 (5,738 to 9,843) | 6,943 (5,255 to 8,783) | 63,951 (52,524 to 76,611) | 79,672 (62,556 to 97,031) | 6,835 (5,318 to 8,508) | 6,897 (5,394 to 8,455) |
| Gujarat | 15,883 (12,180 to 20,797) | 13,245 (10,496 to 16,616) | 74,909 (62,331 to 87,627) | 81,641 (67,442 to 97,226) | 7,090 (5,771 to 8,505) | 6,986 (5,682 to 8,470) |
| Haryana | 11,720 (8,660 to 16,014) | 9,566 (7,594 to 11,898) | 71,773 (59,868 to 83,208) | 76,176 (62,749 to 91,295) | 6,688 (5,525 to 8,010) | 6,959 (5,696 to 8,428) |
| Himachal Pradesh | 9,213 (6,818 to 12,749) | 9,807 (7,620 to 12,392) | 65,388 (56,042 to 75,231) | 92,535 (75,505 to 1,10,842) | 5,996 (4,965 to 7,280) | 8,976 (6,915 to 11,180) |
| Jammu and Kashmir & Ladakh | 10,510 (8,079 to 14,172) | 8,501 (6,757 to 11,029) | 75,756 (65,640 to 87,366) | 89,209 (75,013 to 1,04,297) | 5,814 (4,733 to 7,062) | 7,977 (6,561 to 9,584) |
| Karnataka | 12,086 (8,039 to 18,305) | 10,422 (8,155 to 14,338) | 80,733 (67,221 to 95,078) | 92,851 (76,274 to 1,11,300) | 7,170 (5,783 to 8,696) | 7,564 (6,076 to 9,241) |
| Kerala | 6,699 (4,861 to 9,639) | 6,007 (4,688 to 8,317) | 59,942 (49,907 to 70,580) | 78,042 (64,813 to 93,122) | 5,795 (4,679 to 7,024) | 7,035 (5,388 to 8,586) |
| Maharashtra | 11,192 (8,151 to 15,657) | 9,278 (7,189 to 12,051) | 71,416 (59,907 to 83,558) | 75,114 (61,588 to 90,421) | 6,545 (5,269 to 7,857) | 6,515 (5,215 to 7,829) |
| Manipur | 14,299 (10,290 to 19,299) | 14,353 (10,664 to 18,218) | 67,187 (54,900 to 82,157) | 85,704 (68,400 to 1,03,456) | 5,459 (4,307 to 7,259) | 7,344 (5,698 to 9,301) |
| Meghalaya | 16,989 (12,197 to 22,865) | 17,860 (12,905 to 22,869) | 68,854 (57,011 to 82,004) | 85,303 (68,352 to 1,02,035) | 4,670 (3,677 to 6,222) | 6,405 (4,999 to 8,344) |
| Mizoram | 11,971 (8,700 to 15,603) | 10,620 (7,836 to 13,828) | 67,624 (53,843 to 82,538) | 83,563 (63,807 to 1,03,480) | 5,022 (3,971 to 6,581) | 6,982 (5,493 to 8,533) |
| Nagaland | 13,393 (9,640 to 18,422) | 14,104 (10,187 to 18,567) | 71,386 (57,775 to 86,249) | 85,246 (65,285 to 1,05,831) | 5,525 (4,382 to 6,873) | 7,287 (5,673 to 9,084) |
| Other small union territories | 9,237 (6,724 to 12,576) | 8,771 (6,745 to 11,574) | 63,041 (51,779 to 76,887) | 80,625 (63,576 to 98,466) | 8,856 (6,194 to 11,297) | 7,891 (5,947 to 9,894) |
| Punjab | 9,735 (7,122 to 13,419) | 7,721 (6,098 to 9,707) | 76,414 (63,112 to 88,297) | 69,423 (57,291 to 84,012) | 6,164 (5,002 to 7,324) | 6,517 (5,215 to 7,980) |
| Sikkim | 11,369 (7,917 to 14,851) | 13,165 (8,735 to 16,763) | 66,443 (55,819 to 79,964) | 77,922 (61,593 to 94,306) | 5,140 (4,179 to 6,294) | 7,938 (6,047 to 9,909) |
| Tamil Nadu | 10,920 (7,987 to 15,490) | 10,653 (8,414 to 13,718) | 74,383 (61,554 to 87,902) | 84,263 (68,204 to 1,00,456) | 9,344 (6,595 to 11,656) | 9,718 (6,985 to 12,266) |
| Telangana | 12,059 (7,823 to 20,577) | 9,843 (6,966 to 13,979) | 64,153 (50,687 to 79,959) | 74,171 (57,028 to 93,401) | 7,179 (5,453 to 9,064) | 7,667 (5,995 to 9,890) |
| Tripura | 13,043 (9,476 to 18,096) | 11,397 (8,659 to 15,048) | 74,295 (61,471 to 89,910) | 91,131 (73,036 to 1,08,370) | 4,718 (3,708 to 6,437) | 6,425 (5,060 to 8,179) |
| West Bengal | 11,557 (7,587 to 18,540) | 8,788 (6,404 to 14,927) | 73,124 (59,687 to 86,477) | 71,120 (58,676 to 84,844) | 5,665 (4,628 to 6,795) | 5,577 (4,511 to 6,779) |

# **Supplementary Table 6. Age- and sex- disaggregation in the available** **indicators for services provided to the older persons aged 60 years or more under other relevant national health programs.**

**National Program for Control of Blindness & Visual Impairment (NPCBVI)**

| **Eye care services** | **Disaggregated by age** | **Disaggregated by sex** |
| --- | --- | --- |
| Number of cataract surgeries done | No | No |
| Other eye diseases (diabetic retinopathy, glaucoma, squint, retinopathy of prematurity etc) interventions | No | No |
| Spectacles for near work to elderly persons | No | No |
| Collection of donated eyes | No | No |

**National Program for the Prevention & Control of Deafness (NPPCD)**

| **Services provided** | **Disaggregated by age** | **Disaggregated by sex** |
| --- | --- | --- |
| **Sub Centre/Primary Health Centre** |  |  |
| Number of cases identified with hearing loss | Yes | Yes |
| Number of cases identified with ear discharge | Yes | Yes |
| Number of cases identified with pain in the ear | Yes | Yes |
| Number of cases identified with speech problem | Yes | Yes |
| Number of cases identified with ear trauma | Yes | Yes |
| Number of persons referred to Primary Health Centre/ Community Health Centre | No | No |
| Number of persons referred to District Hospital | No | No |
| Number of persons referred to Medical College | No | No |
| Number of persons referred to Private Doctor | No | No |
| Number of persons referred to any other | No | No |
| **Community Health Centre** |  |  |
| Number of cases identified with hearing loss | Yes | Yes |
| Number of cases identified with ear discharge | Yes | Yes |
| Number of cases identified with pain in the ear | Yes | Yes |
| Number of cases identified with speech problem | Yes | Yes |
| Number of cases identified with wax | Yes | Yes |
| Number of cases identified with ear trauma | Yes | Yes |
| Number of persons treated | No | No |
| Number of persons referred to District Hospital | No | No |
| Number of persons referred to Medical College | No | No |
| Number of persons referred to Private Doctor | No | No |
| Number of persons referred to any other | No | No |
| Reasons for referral |  |  |
| *Surgical Treatment* | No | No |
| *Complications* | No | No |
| *Hearing Aid / Rehabilitation* | No | No |
| *Any other* | No | No |
| **District-level** |  |  |
| Number of cases examined with deafness | Yes | Yes |
| Number of cases examined with mild deafness | Yes | Yes |
| Number of cases examined with moderate deafness | Yes | Yes |
| Number of cases examined with severe deafness | Yes | Yes |
| Number of cases examined with profound deafness | Yes | Yes |
| Number of surgeries performed |  |  |
| *Myringoplasty* | No | Yes |
| *Tympanoplasty* | No | Yes |
| *Myringotomy* | No | Yes |
| *Grommet insertion* | No | Yes |
| *Stapedectomy* | No | Yes |
| *Mastoidectomy* | No | Yes |
| Number of hearing aids fitted | Yes | Yes |
| Number of persons referred for rehabilitation | Yes | Yes |
| **Screening Camps** |  |  |
| Number of patients screened in the camps | No | No |
| Number of cases screened with deafness | Yes | Yes |
| Number of cases screened with mild deafness | Yes | Yes |
| Number of cases screened with moderate deafness | Yes | Yes |
| Number of cases screened with severe deafness | Yes | Yes |
| Number of cases screened with profound deafness | Yes | Yes |
| Chronic suppurative otitis media | Yes | Yes |
| Acute suppurative otitis media | Yes | Yes |
| Secretory otitis media | Yes | Yes |
| Wax | Yes | Yes |
| Ear trauma | Yes | Yes |
| Speech problems | Yes | Yes |
| Any other | Yes | Yes |
| **Medical College** |  |  |
| Number of surgeries performed |  |  |
| *Myringoplasty* | No | Yes |
| *Tympanoplasty* | No | Yes |
| *Myringotomy* | No | Yes |
| *Grommet insertion* | No | Yes |
| *Stapedectomy* | No | Yes |
| *Mastoidectomy* | No | Yes |
| Number of hearing aids fitted | Yes | Yes |
| Number of persons medically rehabilitated | Yes | Yes |
| Number of persons referred for educational/vocational rehabilitation | Yes | Yes |

**National Mental Health Program (NMHP)**

| **Outpatient Department (OPD) and referral services at the District Health Care Level** | **Disaggregated by age** | **Disaggregated by sex** |
| --- | --- | --- |
| Total number of new patients seen in the OPD in the reported quarter | No | No |
| Total number of follow-up cases seen in the OPD in the reported quarter | No | No |
| Total number of cases referred to tertiary care hospital in the reported quarter | No | No |
| **Inpatient Department (IPD) at the District Health Care Level** |  |  |
| Total number of patients admitted in IPD | No | No |
| Average duration of stay in days | No | No |
| **After treatment continuing care services at the District Level** |  |  |
| Total number of patients availed services at Day Care Centres | No | No |
| Total number of patients availed services at Residential Continuing Care Centres | No | No |
| Total number of patients availed services at Long Term Residential Continuing Care Centres | No | No |
| **Outreach services** |  |  |
| Total number of cases examined in the outreach camps | No | No |
| Total number of cases referred at District Level for management | No | No |
| Total number of cases referred for rehabilitation/counselling | No | No |

| **Outpatient Department (OPD) and referral services at the Primary Health Centre Level** | **Disaggregated by age** | **Disaggregated by sex** |
| --- | --- | --- |
| Total number of new patients seen in the OPD in the reported quarter | No | No |
| Total number of follow-up cases seen in the OPD in the reported quarter | No | No |
| Total number of cases referred to District Hospital in the reported quarter | No | No |
| Total number of patients referred for counselling services | No | No |
| Total number of cases referred back from the District Level for follow-up treatment | No | No |
| **Outpatient Department (OPD) and referral services at Community Health Centre Level/Taluk Hospital Level** |  |  |
| Total number of new patients seen in the OPD in the reported quarter | No | No |
| Total number of follow-up cases seen in the OPD in the reported quarter | No | No |
| Total number of cases referred to District Hospital in the reported quarter | No | No |
| Total number of patients referred for counselling services | No | No |
| Total number of cases referred back from the District Level for follow-up treatment | No | No |
| **Inpatient department (IPD) at Community Health Centre Level/Taluk Hospital Level** |  |  |
| Total number of patients admitted in IPD | No | No |
| Average duration of stay (in days) | No | No |

**National Program for Prevention and Management of Osteoarthritis & other Musculoskeletal Disorders (MSDP)**

| **Services provided** | **Disaggregated by age** | **Disaggregated by sex** |
| --- | --- | --- |
| Total number of persons attended the outreach camps | No | No |
| Total number of persons attended the outreach camps during the reporting period | No | No |
| Total number of patients visited health facilities for musculoskeletal problems | No | No |
| Number of patients who turned up for follow up | No | No |
| Number of patients with improvements in signs and symptom | No | No |
| Number of patients with improvements in the quality of life with respect to pain, range of movement by goniometer, WOMAC scale, vas score, activity, sleep and anxiety | No | No |
| Number of patients with reduction in the intake of allopathic medicines | No | No |

|  | Age disaggregated data available as 0-5 years, 6-15 years, 16-50 years, and 50 years or more |
| --- | --- |
|  | Age/sex disaggregated data available |
|  | Age/sex disaggregated data not available |

# **Supplementary Table 7. Age- and sex- disaggregation in the available** i**ndicators for monitoring services for older persons aged 60 years or more at the Health and Wellness Centres (HWCs).**

| **Indicators** | **Disaggregated by age** | **Disaggregated by sex** |
| --- | --- | --- |
| Percent of elderly registered at the HWCs | No | No |
| Percent of elderly population screened as a part of Comprehensive Geriatric Assessment by Community Health Officer | No | No |
| Percent of elderly on treatment at HWC provided cross referral to Community Health Centres/District Hospitals/Regional Geriatric Centres | No | No |
| Percent of elderly provided physiotherapy services | No | No |
| Percent of home-bound (bed-bound and restricted mobility) elderly visited by Accredited Social Health Activist & Auxiliary Nurse and Midwife/ Multipurpose Health Worker-Male | No | No |
| Percent of needy elderly provided with supportive/assistive devices | No | No |
| Percent of single elderly (elderly living alone) visited by Accredited Social Health Activist and Multipurpose Health Worker | No | No |
| Number of elderly support groups- “Sanjeevini” created | No | No |

|  | Age/sex disaggregated data available |
| --- | --- |
|  | Age/sex disaggregated data not available |
